# Bispecific Antibodies: From Research to Clinical Application

**DOI:** 10.3389/fimmu.2021.626616

**Published:** 2021-05-05

**Authors:** Jiabing Ma, Yicheng Mo, Menglin Tang, Junjie Shen, Yanan Qi, Wenxu Zhao, Yi Huang, Yanmin Xu, Cheng Qian

**Affiliations:** ^1^ College of Life Sciences and Medicine, Zhejiang Sci-Tech University, Hangzhou, China; ^2^ IND Center, Chongqing Institute of Precision Medicine and Biotechnology Co., Ltd., Chongqing, China; ^3^ IND Center, Chongqing Precision Biotech Co., Ltd., Chongqing, China; ^4^ Center for Precision Medicine of Cancer, Chongqing Key Laboratory of Translational Research for Cancer Metastasis and Individualized Treatment, Chongqing University Cancer Hospital, Chongqing, China

**Keywords:** BsAbs, development, platform, mechanism, application

## Abstract

Bispecific antibodies (BsAbs) are antibodies with two binding sites directed at two different antigens or two different epitopes on the same antigen. The clinical therapeutic effects of BsAbs are superior to those of monoclonal antibodies (MoAbs), with broad applications for tumor immunotherapy as well as for the treatment of other diseases. Recently, with progress in antibody or protein engineering and recombinant DNA technology, various platforms for generating different types of BsAbs based on novel strategies, for various uses, have been established. More than 30 mature commercial technology platforms have been used to create and develop BsAbs based on the heterologous recombination of heavy chains and matching of light chains. The detailed mechanisms of clinical/therapeutic action have been demonstrated with these different types of BsAbs. Three kinds of BsAbs have received market approval, and more than 110 types of BsAbs are at various stages of clinical trials. In this paper, we elaborate on the classic platforms, mechanisms, and applications of BsAbs. We hope that this review can stimulate new ideas for the development of BsAbs and improve current clinical strategies.

## Introduction

Various factors and multiple signaling pathways are involved in cancers and other complex diseases. Therefore, it is difficult to obtain satisfactory with MoAbs for drug resistance, and most studies on MoAb combination therapies are still in the early stage (1). Compared with MoAbs, BsAbs offer more advantages. In terms of superior cytotoxic effects, under tumorigenic conditions and infections, there is a lower rate of resistance to them due to the matched targeting of two different antigens (2). Since the original concept of BsAbs was first proposed by Nisonoff and his collaborators in the 1960s (3, 4), the first insight into antibody architecture, followed by numerous other finding, have been reported. In 1975, the invention of hybridoma technology finally solved the problem of producing pure antibodies, which marked the arrival of a new era of MoAbs therapy (5). In 1983, hybrid-hybridoma (quadroma) technology was pioneered by Milstein and Cuello (6). Then, in 1988, the Huston team invented the single-chain variable fragment (scFv), which has minimized the refolding problems, such as incorrect domain pairing or aggregation, of two-chain species (7). However, it was not until the knobs-into-holes technology emerged in 1996 that BsAbs became more developed (8). Subsequently, with progress in antibody engineering and antibody biology, the concept and technology of constructing BsAbs continued to evolve. In addition to the development of various platforms, the applications of BsAbs are diverse and the potential combination of targets is flexible. This review provides a comprehensive and systematic summary of the classic platforms, mechanisms, and applications of BsAbs.

## BsAb Molecular Platforms

The main challenge encountered in the development of BsAbs is that there are two types of chains, heavy and light, that when mismatched may produce a variety of side products (9–11). Therefore, several strategies are used to achieve correct matching of heavy and light chains. BsAbs are usually divided into two types: IgG-like and non-IgG-like. The relatively large molecular weight of IgG-like BsAbs helps to purify and improve solubility and stability, increase the serum half-life and affinity, and thereby enhance biological activity (12). Non-IgG-like BsAbs only have therapeutic effects through antigen binding because of the lack of Fc fragments. They are easy to produce and have low immunogenicity (13). Further studies on the correct matching of heavy chains are needed for IgG-like BsAbs than for non-IgG-like BsAbs because of the presence of Fc. The following sections introduce some of the BsAb molecular platforms.

### Platforms of IgG-Like BsAbs

The heavy chain in IgG-like BsAbs has been modified to promote heterologous Fc matching. For example, knobs-into-holes changes the local spatial structure of Fc (14, 15). The strand-exchange engineered domain (SEED) platform uses complementary sequences to promote heterodimer assembly (16). Whereas the DEKK platform uses mutations to form salt bridges (17). Orthogonal Fab (18), ART-Ig (19), and FAST-Ig depends on electrostatic manipulation for interactions. The DuoBody platform controls Fab dynamic recombination exchange (20). Dual variable domain immunoglobulin (DVD-Ig) and Fabs-in-tandem immunoglobulin (FIT-Ig) platforms are symmetrical structures (21–23). Two-in-one and other molecular platforms rely on phage display and use Crossmab and Wuxibody molecular platforms for light chains to promote correct matching (24–27).

#### Knobs-Into-Holes

The knobs-into-holes model is a novel and effective design for engineering antibody heavy chain homodimers for heterodimerization. The principle behind knobs-into-holes is based on replacing a smaller amino acid with a larger amino acid (T336Y) in the CH3 region of an antibody chain to form a “knobs” structure, and at the same time substituting a larger amino acid in the other chain with a smaller amino acid to form a “holes” structure (Y407T). This structure has a recombination efficiency of 57% (15). Subsequently, Merchant et al. developed a tactic for constructing human BsIgG that greatly eliminates mispairing between light chains and heavy chains. Moreover, they evaluated the effect of v1–v16 variants on the yield of Ab/IA hybrid. The mutations S354C/Y349C and Y349C/E356C are critical factors affecting the production of Ab/IA for variant v8. In addition, v11, v13, and v14 variants, which are cysteine replacement mutations, further increase Ab/IA yield. As the most potent variant, v11 (S354C: T366W/Y349C: T366S: L368A: Y407V) greatly improved the heterodimerization ratio up to a maximum of approximately 95% (8).

#### SEED

Heterologous recombination of the Fc segment can also be achieved *via* the SEED molecular platform. The alternating sequence of human IgA and IgG in the SEED CH3 domain produces two asymmetric but complementary domains called AG and GA. The SEED design allows efficient generation of AG/GA heterodimers and is not conducive to the formation of homodimers between AG and GA. Simultaneously, SEED can be combined with Fab, ScFv, and VHH structures (28). SEED fusion retains complete antibody binding affinity and has excellent biochemical and biophysical stability (29). Intravenous injection in mice showed that SEED produces pharmacokinetics with a longer serum half-life (16).

#### DEKK

The DEKK platform generates both L351D and L368E mutations in one of the heavy chains and simultaneously generates L351K and T366K mutations in the other heavy chain. The IgG main chain can accommodate the introduction of lysine side chains to form a stable salt bridge interaction with the opposite chain (30). MCLA-128 is based on a DEKK platform that targets HER2 and HER3 (17). MCLA-128 has shown good efficacy in clinical trial (phase II: NCT03321981) in patients with breast cancer metastasis and in another clinical trial (phase I/II: NCT02912949) in patients with pancreatic cancer and non-small cell lung cancer(NSCLC) fused with NRG1 (31). The pharmacokinetics of MCLA-128 exhibit similar disposition characteristics to those of other therapeutic MoAbs, and the impact of body-size parameters on the disposition of MCLA-128 has been found to be minimal (32).

#### ART-Ig

The ART-Ig platform promotes heterodimer recombination by introducing different charges in the Fc region (33). If one chain introduces (D360K, D403K) mutations, then the other chain introduces (K402D, K419D) mutations. Or if one chain introduces (T394D, P395D, P396D) mutations, then the other chain introduces (P395K, P396K, V397K) mutations. ERY947 is a BsAb based on the ART-Ig molecular platform with a common light chain targeting GPC3 and CD3. It shows high effectiveness against GPC3-expressing tumors with controllable and reversible cytokine release (34). Emicizumab is also based on this platform. A non-common light chain FAST-Ig molecular platform that introduces different charges in CH1 and CL has been developed on the basis of this platform. Based on this FAST-Ig platform, NXT007 targets FIXa/FX to exert more significant effects in treating hemophilia than emicizumab does (35).

#### Orthogonal Fab

The principle of this platform is the introduction of mutations to generate an “orthogonal interface” that enables preferential alignment of the different Fab domains with correct assembly (12). VRD1 (VL-Q38D VH-Q39K/VL-D1R VH-R62E) mutations and CRD2 (CL-L135Y S176W/CH1-H172A F174G) mutations are introduced in the variable region of one antibody, and the VRD2 (VL-Q38R VH-Q39Y) mutation is introduced into the region of another antibody to reduce light chain mismatches. By using this platform, BsAbs can be stably expressed in mammalian cells (18). LY3164530, which targets EGFR and c-mesenchymal-epithelial transition factor (c-MET) also uses this platform and a phase I (NCT02221882) study in patients with advanced and metastasis cancer is on-going (36). In cell-based assays, LY3164530 has been shown to exhibit superior activity in overcoming HGF-mediated resistance to erlotinib, gefitinib, lapatinib, or vemurafenib compared with the combination of individual monoclonal antibodies targeting these receptors (37).

#### DuoBody

The Fab arm of the IgG4 antibody can be dynamically recombined and exchanged *in vivo* to formed BsAbs, in a process referred to as named Fab-arm exchange (FAE) (38–40). Controlled Fab-arm exchange (cFAE) is the core technology of the Duobody platform. The Duobody platform is only necessary for promoting the completion of the Fab-arm exchange between the two antibodies to form a BsAb by introducing K409R and F405L mutation sites in the CH3 regions (41). The current BsAbs produced by this molecular platform are JNJ-63709178 (phase I: NCT02715011), which targets CD3 and CD123 for AML and JNJ-61186372 (phase I: NCT02609776), which targets EGFR and c-MET for NSCLC. JNJ-61186372 blocks ligand-induced EGFR and c-MET phosphorylation, and more effectively inhibits ERK and AKT phosphorylation and downstream signal activation (42, 43). DuoBody-CD3xCD20 has shown specific and highly potent preclinical anti-tumor activity in lymphoma models *in vitro* and *in vivo* (44).

#### DVD-Ig and FIT-Ig

DVD-Ig and FIT-Ig molecular platforms are symmetrical with four antigen binding sites that can target two different targets at the same time. The difference is that DVD-Ig only contains a pair of Fab domains while the FIT-Ig structure contains two pairs of Fab domains. The DVD-Ig platform contains the Fc region, and each antibody arm uses flexible short peptides to connect two variable regions (45, 46). Antibodies that use the DVD-Ig platform are ABT122 (IL-7×TNF-α), which is used to treat patients with rheumatoid arthritis (RA) who have experienced an inadequate response to methotrexate (47); and ABT165 (Delta-like ligand 4, DLL4×VEGF) which can effectively and significantly inhibit tumor growth in U87-MG human glioblastoma and SW480 human colon cancer xenograft models. ABT-165 induces a stronger anti-tumor response in combination with chemotherapeutics, and has been proven to show good efficacy and safety *in vivo (* 48). The FIT-Ig platform was developed in 2017 (22). EpimAb has developed EMB01, using the FIT-Ig platform, to concurrently target both EGFR and c-MET in order to treat patients with advanced/metastatic solid tumors, including but not limited to NSCLC, colorectal cancer (without RAS positive mutations), gastric cancer, and liver cancer. Compared with anti EGFR mAb or anti c-MET mAb alone, EMB-01 induces significantly stronger anti-tumor activity in PDX and CDX tumor models (49).

#### DAF

This platform uses phage display technology to optimize the herceptin antibody with changes to 11 residues in VL CDRs to specifically bind to VEGF while retaining the ability to bind to HER2, thus achieving the binding on a single antibody can simultaneously to two targets (24). Therefore, this platform is also called the two-in-one platform or dual action fab (DAF) platform (21). MEHD7945A simultaneously targets HER3 and EGFR, and inhibits their downstream signal transduction. Compared with the monospecific anti-HER3 antibody, MEHD7945A has a wider range of efficacy in a variety of tumor models, such as pancreatic cancer, NSCLC and carcinoma of the head and neck (50, 51).

#### CrossMab and Wuxibody

Crossmab and Wuxibody are both used for resolving the BsAb light chain mismatch. The difference between them is that Crossmab resolves the light chain mismatch by exchanging one side of CL and CH1, while WuxiBody introduces a T cell receptor (cα and cβ) replace CL and CH1 on one side.

The CrossMab platform is mainly optimized for light chains. By swapping the regions of one side heavy chain and light chain, the BsAb light chain can be assembled correctly. It is usually combined with other platforms such as knobs-into-holes, DEEK, and ART-Ig. The process of light and heavy chain exchange can be divided into Fab exchange, variable region exchanges, and constant region exchange. Fab regions exchange produces no functional side products. Variable regions exchange produces Bence-Jones-like side products (52, 53). It has been shown that exchanging the CH1 of the heavy chain with the CL of the light chain is more effective, and this is currently used as a common method (54–56).

With the utilization of the CrossMab platform, in addition to bivalent BsAbs structures, trivalent and tetravalent BsAbs structures can also be generated. A large variety of customized BsAbs based on the CrossMab platform have entered the clinic research (55). Vanucizumab (RG7221) targets ANG-2/VEGF, in which the correct matching of the heavy chain region is achieved *via* the knobs-into-holes platform (57). VEGF/Ang-2 (RG7716) is the first BsAb to be used in an ophthalmology clinical study. CD3×CEA (RG7802) targets CEA positive solid tumors (58), and FAP-DR5 (RG7386) recognizes fibroblast activation protein α (FAP) and death receptor 5 (DR5) on tumor cells (59).

The Wuxibody platform is created by WuXi Biologics. It is based on the heterodimer of TCR, which replaces the CL and CH1 of the antibody with the constant region of the TCR, so that the light chain of the antibody can be matched correctly (60). The tethered-variable CL BsIgG (TcBsIgG) platform, which was developed by Genentech, also promotes the correct matching of the light chain. The antibodies VL and VH are connected with (G4S)4; then the CL and the two heavy chains are expressed together to obtain the correct BsAb (61).

### Platforms of Non-IgG-Like BsAbs

The design of non-IgG-like antibodies is relatively simple because of the lack of the Fc segment. For example, bispecific T-cell engager (BiTE) uses linker to connect two ScFvs (62). The Fv of dual affinity retargeting (DART) is formed by the association of a VL partner on one chain with a VH partner on the second chain (63, 64). Other platforms involve tetravalent antiparallel structure (TandAbs) and VH only (Bi-Nanobody) (65–68). Although non-IgG-like BsAbs have high tumor tissue permeability because of their low molecular weight, they a relatively short half-life and require multiple doses (10, 13, 69).

#### BiTE

BiTE connects the CD3 antibody ScFv and the tumor-associated antigen (TAA) or tumor-specific antigen ScFv with G4S linker to redirect T cells to cancer cells and perform targeted killing (70, 71). In addition to blinatumomab, other BsAbs use the BiTE platform; these include CD3 × BCMA (B-cell maturation antigen) (AMG420) (72, 73), CD3 × CD33 (AMG330) (74, 75), CD3 × PSMA (prostate-specific membrane antigen) (AMG212) (76), and CD3 × EGFRvIII (epithelial growth factor receptor variant III) (AMG596) (77, 78). BsAbs on the BiTE platform are small and have a short half-life of only about 2 h which means necessitates administration with continuous intravenous infusion is needed (79, 80). Based on the BiTE platform, Amgen developed the second-generation BiTE platform, known as half-life extended BiTE (HLE-BiTE), which connects Fc to the end of ScFv extends its half-life to 7 days (81). CD3×CD19(AMG562), CD3×CD33(AMG673) (82), CD3×FLT3(FMS-like tyrosine kinase 3)(AMG427) (83), and CD3×BCMA(AMG701) all utilize the HLE-BiTE platform (84).

The combination of BiTE and chimeric antigen receptor T cell (CAR-T) immunotherapy can be applied in cancer treatment, there is an example of using BiTE platform BsAbs and CAR-T immunotherapy. EGFRvIII is overexpressed in glioblastoma cells. Elimination of EGFRvIII-positive tumor cells by CAR-T cells results in a loss of the target EGFRvIII, which consequently leads to relapse. When the BsAb sequence is added after the EGFRvIII CAR-T plasmid sequence, CAR-T cells can target EGFRvIII-positive tumor cells, and the released BsAb can recruit T cells to form a bridge, thereby killing EGFR-positive tumor cells that have lost the EGFRvIII antigen. The combination of CAR-T immunotherapy and BsAb therapy can lead to a better tumor remission than the use of either therapy alone (85).

#### DART

The DART platform is formed by linking VH and VL sequences with other antibody VL and VH sequences, respectively. In addition, cysteine is introduced at the C-terminus of the two polypeptide chains to form an interchain disulfide bond. Flotetuzumab (MGD006), which targets CD3 and CD123, is based on a DART platform for relapsed or refractory acute myeloid leukemia and myelodysplastic syndrome (86, 87). An cynomolgus monkey model showed that flotetuzumab was well tolerated. Of 14 patients treated in clinical trials (phase I/II: NCT02152956), 28% achieved CR. The most common side effects were infusion-related reactions and cytokine release syndrome (CRS) (87, 88). MGD007, another DART platform BsAb, targets CD3 and GPA33 for GPA-positive colorectal cancer (89). In comparison with the BiTE designs, DART achieves a higher magnitude of T cell activation (63).

#### TandAbs

The TandAbs platform is a tetravalent antibody molecule with two binding sites for each of two antigens (67). A homodimer molecule is formed by the reverse pairing of two peptide chains. AFM11, which targets CD3 and CD19, is based on the TandAbs platform and has more significant and marked therapeutic effects. In a xenograft model, AFM11 showed dose-dependent inhibition of the growth of Raji tumors *in vivo* as well as good tumor localization (66).

#### Bi-Nanobody

**Figure 1:**
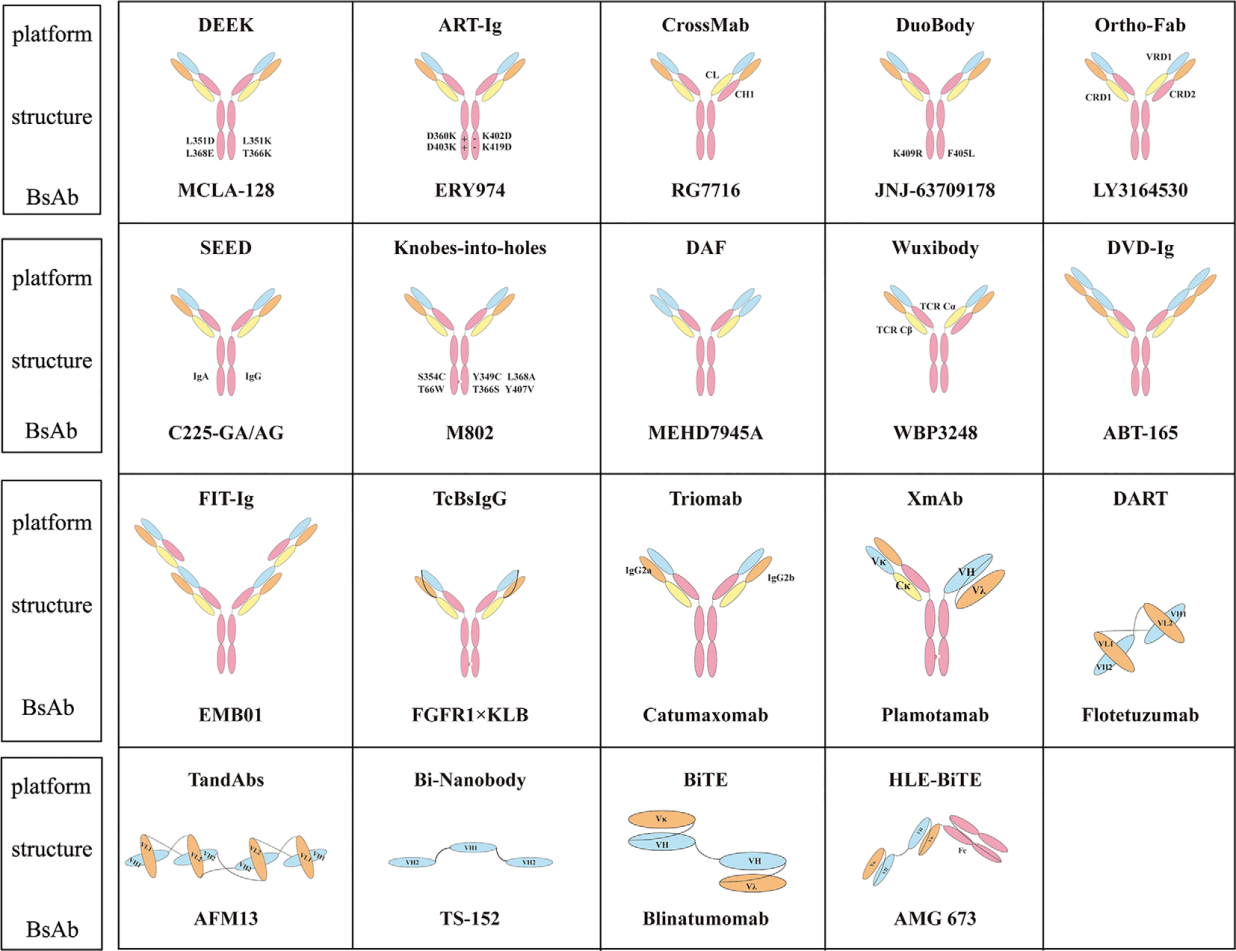
Classical molecular platform of BsAbs and representative antibodies.

The bi-Nanobody platform connects the VH regions of two or more antibody molecules to achieve multi-specific binding. The products of this platform are small molecules that possess the advantage of high stability and better tissue permeability *in vivo (* 90). Ozoralizumab (ATN-103, TS-152), developed by Ablynx, is a trivalent BsAb targeting albumin (ALB) and tumor necrosis factor (TNF) for RA. Currently, ozoralizumab is being test in five clinical trials, one of which has reached phase III (NCT04077567). Ablynx has also developed another BsAb to treat RA, vobarilizumab (ALX-0061), which targets ALB and IL6R. After a single intravenous injection of ALX-0061 in cynomolgus monkeys, the half-life in plasma was 6.6 days, which was similar to the expected half-life of serum albumin. In nonhuman primate models, serum c-reactive protein levels were significantly reduced (91). **Figure 1** summarizes the structure of BsAbs on different platforms.

## Mechanisms of Action of BsAbs

Because BsAbs have two binding sites directed at different antigens or recognize two different epitopes of one antigen simultaneously, the functioning pathways are quite flexible. Some BsAbs play the role of immune cell connector, connecting immune cells to tumor cells and enabling immune cells to exert their killing effect of immune cells. Most BsAbs target CD3, CD16, and CD47, and some release immune cells from an immunosuppressed state for dual-targeted immune checkpoints, such as PD1, CTLA-4, LAG-3 (92, 93). Some BsAbs target the immune checkpoint and TAAs, while other BsAbs target CD28 and CD137 to activate immune cell activity (94). In addition to targeting immune cells, BsAbs target tumor antigens, blocking dual signaling pathways. The main targets are PSMA, HER2, HER3, EGFR, DLL1, and ANG-2. Besides targeting dual tumor targets, some target inflammatory factors in the tumor microenvironment to reduce inflammation and CRS. Most BsAbs target tumors, although some are used to treat hemophilia A, neovascular age-related macular degeneration, diabetes, pneumonia caused by bacteria, and Alzheimer’s disease.

### Recruitment and Activation of Immune Cells

CD3 is a characteristic antigen expressed on the surface of T cells. By targeting the CD3 antigen and a TAA, BsAbs can redirect T cells to cancer cells to exert a killing effect (95). Catumaxomab and blinatumomab are examples of antibodies in the market that recruit and activate immune cells. However, because catumaxomab is a murine antibody as well as an overly high-affinity antibody of CD3 (4.4 nM), T cells are overactivated, leading to CRS. AMG 424, designed by Amgen, was based on the XmAb platform which heterologous recombination from ScFv-Fc of CD3 and Fab-Fc of CD38 (96). CD38 is highly expressed in malignant multiple myeloma cells, but weakly expressed in lymphocytes and NK cells (97). Therefore, while CD38 is not an ideal target for CAR-T immunotherapy, AMG 424 has shown great therapeutic potential in animal models. AMG 424 uses the low-affinity CD3 antibody instead of the high-affinity CD3 antibody; the release of TNF-α and IFN-γ is reduced, but the effect is not changed. Furthermore, AMG 424 inhibits tumor growth in bone marrow, and thus it is the only BsAb drug approved by the FDA to treat multiple myeloma (98).

In addition to T cells, specific antigens on the surface of NK cells and macrophages are often used as targets for BsAbs. AFM13, which targets CD16A and CD30, is a tetravalent BsAb based on the TandAbs platform used to treat malignant tumors that express CD30. AFM13 recruits NK cells to exert killing effects against CD30-positive Hodgkin’s lymphoma and has low cytokine release (99). AFM13 is used to treat Hodgkin’s lymphoma. Data from a phase I study (NCT01221571) of severe relapsed or refractory Hodgkin’s lymphoma showed that AFM13 has a half-life up to 19 h; three of 26 evaluable patients achieved partial remission (11.5%) and 13 patients achieved stable disease progression (50%), the overall disease control rate was 61.5% (100). In a phase 1b study (NCT02665650), the remission rate for AFM13 combined with pembrolizumab reached 88% at the highest therapeutic dose. The ORR was 83%. A pharmacokinetic evaluation of AFM13 in combination therapy showed that its half-life extended to 20.6 h (101), demonstrating an excellent clinical effect. CD47 is often expressed on the surface of tumor cells; CD47-SIRPα releases a “don’t eat me” signal to DC cells, thus evading recognition by DC cells. NI-1801 targets CD47 and mesothelin, and NI-1701 targets CD47 and CD19 to enhance the phagocytotic effect of DC cells. NI-1801/NI-1701 depends on the κλ-body platform, which uses the same heavy chain, two different light chains, one κ chain, and one λ chain. It is a fully human bispecific IgG without any modification, with high stability and a long half-life (102, 103).

### Blocking of Immune Checkpoints

Immune checkpoints, such as PD1, CTLA-4, LAG-3, have an inhibitory effect on the activity of immune cells (104). When these checkpoints are blocked with antibodies, immune cells can be reactivated. BsAbs targeting immune checkpoints are mostly indicated for solid tumors, such as AK104, MGD019, XmAb20717, MEDI5752, MGD013, RO7121661, which are dual targeting immune checkpoints. The functions of lymphocytes co-expressed by PD-1 and LAG-3 are mostly dysregulated, and combined treatment with PD-1 and LAG-3 can effectively restore T cell function (105). MGD013 is a tetravalent DART BsAb. As of April 25, 2020, the overall safety of MGD013 (including the incidence of immune-mediated adverse events) was generally consistent with PD-1 antibody monotherapy in terms of event type and frequency, whereas MGD013 had better efficacy. BsAbs of immune checkpoints combined with TAAs, such as AK112 (PD1×VEGF) and IBI315 (PD1×HER2), have also been developed.

### Blocking of Inflammatory Factors

RA is a chronic inflammatory autoimmune disease of the joints. Synovial inflammation, cartilage damage, and bone erosion are the main symptoms of RA (106). Antibodies targeting proinflammatory factors TNF, IL-1β, IL-23, IL-17, IL-6, can treat RA (107). However, some patients do not respond to TNF-α. In these patients, IL-17A concentration increases and is positively correlated with the severity of the disease. Preclinical and clinical studies have shown that blocking two or more cytokines can be more efficacious than using MoAbs alone (108). ABT122, based on the DVD-Ig platform, targets TNF-α and IL-17 dual factors to treat RA. In addition, ABT-122 can quickly alter the potential pathophysiological pathways of RA patients (including reducing the chemokines CXCL9, CXCL10, and CCL23) and completely inhibit the production of IL-6 induced by TNF-α and IL-17A (109–111). RO7040547 targets IL-17 and IL-13. SAR156597 targets IL-4 and IL-13. In the CD40-induced colitis model, BsAbs target IL-23 and TNF-α shows a synergistic effect relative to antibodies against TNF-α and IL-23 alone in inflammatory bowel disease. Targeting IL-23 and IL-6, AZ17 as a BsAb in the preclinical development stage, is theoretically possible to treat inflammatory bowel disease (112, 113).

### Blocking of Dual Signaling Pathways

BsAbs can target two specific antigens or different areas of the same antigen at the same time (114). Blocking signal transmission along signal pathways can affect the occurrence and development of tumors, target the tumor microenvironment, and inhibit the regeneration of tumor blood vessels. For example, BI 836880 and vanucizumab target EGFR and ANG2; ABT-165 targets DLL4×VEGF, OMP-305B83, and TR009; EMB01 targets EGFR and c-MET; and MCLA-158 targets EGFR×LGR5. ZW25 simultaneously targets different epitopes of HER2.

HER2, also known as neu, ErbB-2, CD340, and p185, is a member of the ERBB family of receptor tyrosine kinases. This family also includes ERBB1, ERBB3, and ERBB4. HER2 does not bind directly to ligands but can achieve heterodimerization with any other EGFR family receptor (115). It is the preferred heterodimerization partner for all ERBB proteins (116). Heterodimerization enhances signal transmission and initiates several downstream signaling pathways (117), including MAPK, PI3K/AKT/mTOR, SRC, and STAT signal transduction cascades, leading to cell proliferation, migration, and differentiation (118–120). The structure of HER2 includes an N-terminal extracellular domain, a transmembrane domain, and an intracellular domain. The extracellular domain comprises four domains. Trastuzumab, which targets HER2 extracellular domain IV, is used to treat metastatic breast cancer in patients overexpressing HER2 (121). Pertuzumab targets HER2 extracellular domain II, thereby inhibiting dimerization between HER receptors (122). ZW25 targets HER2 extracellular domains II and IV simultaneously. ZW25 blocks the downstream signal transmission of HER2 and has good tolerability and considerable single-dose activity (123). In one study, the ORR of patients (most with gastroesophageal cancer or colorectal cancer) was 41%, and the side effects were mild (124).

EMB-01 is generated from FIT-Ig, targeting both c-MET and EGFR to treat NSCLC (49). The EGFR antibody inhibits activation of the EGFR signaling pathway by blocking the binding of ligands and receptors. However, EGFR gene mutations and c-MET gene amplification cause EGFR antibody resistance. On the one hand, EMB-01 can prevent ligands from activating the EGFR and c-MET pathways; on the other hand, EMB-01 molecules can bind two EGFR molecules and c-MET molecules at the same time to form a complex structure on the cell surface, thereby inducing endocytosis. This process, which is irreversible, can fundamentally eliminate these two receptors on the surface of tumor cells, and achieve long-term effects. **Table 1** summarizes some clinical BsAbs and their mechanisms of action.

**Table 1:** Summary of clinical BsAbs and their mechanisms of action.

BsAbs mechanisms of action	Name	Target 1	Target 2	Platform	R & D institutions	Conditions	Phase (NCT)	Study Start	Status
**Recruitment and activation of immune cells**	MGD011	**CD3**	CD19	DART	MacroGenics	B cell lymphoma	**phase II** (NCT03635593)	January 1, **2019**	Not yet recruiting
	AFM11		CD19	TandAbs	Affimed	NHL	**phase I **(NCT02848911)	October **2016**	Terminated
	Blinatumomab		CD19	BiTE	Amgen	ALL	**Approved**	NA	Approved
	AMG562		CD19	HLE-BiTE	Amgen	Lymphoma	**phase I** (NCT03571828)	October 29, **2018**	Active, not recruiting
	A-319		CD19	ITab	Generon	ALL	**phase I **(NCT04056975)	September 15, **2019**	Not yet recruiting
	RG7828		CD20	KIH	Roche	Hematological malignancies	**phase I/II** (NCT03677141)	February 8, **2019**	Recruiting
	REGN1979		CD20	NA	Regeneron	Hematological malignancies	**phase II **(NCT03888105)	November 13, **2019**	Recruiting
	RG6026		CD20	CrossMab/KIH/TCB	Roche	NHL	**phase I **(NCT03075696)	February 21, **2017**	Recruiting
	GEN3013		CD20	DuoBody	Genmab	Hematological malignancies	**phase I/II** (NCT03625037)	June 26, **2018**	Recruiting
	FBTA05		CD20	Triomab	Trion	B cell lymphoma	**phase I/II** (NCT01138579)	August **2010**	Terminated
	Plamotamab		CD20	Xmab	Xencor	Hematological malignancies	**phase I** (NCT02924402)	October **2016**	Recruiting
	AMG330		CD33	BiTE	Amgen	AML	**phase I **(NCT04478695)	September 18, **2020**	Not yet recruiting
	AMG673		CD33	HLE-BiTE	Amgen	AML	**phase I** (NCT03224819)	September 7, **2017**	Recruiting
	AMV-564		CD33	TandAb	Amphivena Therapeutics	AML	**phase I **(NCT04128423)	October 9, **2019**	Recruiting
	GEM333		CD33	scDb	GEMoaB Monoclonals	AML	**phase I** (NCT03516760)	April 11, **2018**	Active, not recruiting
	GBR 1342		CD38	BEAT	Glenmark Pharmaceuticals	MM	**phase I/II **(NCT03309111)	October 16, **2017**	Recruiting
	AMG424		CD38	Xmab	Amgen	MM	**phase I** (NCT03445663)	July 31, **2018**	Terminated
	AMG420		BCMA	BiTE	Amgen	MM	**phase I** (NCT03836053)	March 4, **2019**	Active, not recruiting
	AMG701		BCMA	HLE-BiTE	Amgen	MM	**phase I** (NCT03287908)	November 13, **2017**	Recruiting
	JNJ-64007957		BCMA	DuoBody	Janssen	MM	**phase I** (NCT03145181)	May 16, **2017**	Recruiting
	EM801		BCMA	CrossMab/KIH	Celgene	MM	**phase I** (NCT03486067)	April 3, **2018**	Recruiting
	PF-06863135		BCMA	NA	Pfizer	MM	**phase I** (NCT03269136)	November 29, **2017**	Recruiting
	REGN5458		BCMA	NA	Regeneron	MM	**phase I/II** (NCT03761108)	January 23, **2019**	Recruiting
	TNB-383B		BCMA	NA	AbbVie	MM	**phase I** (NCT03933735)	June 24, **2019**	Recruiting
	APVO436		CD123	(scFv-Fc)2	Aptevo Therapeutics	AML	**phase I** (NCT03647800)	December 13, **2018**	Recruiting
	MGD006		CD123	DART	MacroGenics	AML	**phase II** (NCT03739606)	April 10, **2020**	Not yet recruiting
	Xmab14045		CD123	Xmab	Xencor	AML/CML	**phase I** (NCT02730312)	August **2016**	Recruiting
	JNJ-63709178		CD123	DuoBody	Janssen	AML	**phase I** (NCT02715011)	June **2016**	Recruiting
	MCLA-117		CLEC12A	Biclonics	Merus	AML	**phase I** (NCT03038230)	April **2016**	Unknown
	RG6160		FcRH5	BiTE	Genentech	MM	**phase I** (NCT03275103)	September 19, **2017**	Recruiting
	AMG427		FLT3	HLE-BiTE	Amgen	AML	**phase I** (NCT03541369)	September 14, **2018**	Recruiting
	JNJ-64407564		GPRC5D	DuoBody	Janssen	MM	**phase I** (NCT03399799)	December 16, **2017**	Recruiting
	AMG111		CEA	BiTE	Amgen	Gastrointestinal adenocarcinoma	**phase I** (NCT01284231)	December **2010**	Completed
	RG7802		CEA	CrossMAb	Roche	Solid tumor	**phase I** (NCT02650713)	January 7, **2016**	Completed
	Solitomab		EpCAM	BiTE	Amgen	Malignant ascites	**phase I** (NCT00635596)	March **2008**	Completed
	Catumaxomab		EpCAM	Triomab	Trion	Malignant ascites	Approved	NA	Approved
	Pasotuxizumab		PSMA	BiTE	Bayer	Prostate cancer	**phase I** (NCT02806973)	January **2015**	Completed
	HPN-424		PSMA	DART	Harpoon	Prostate cancer	**phase I/II** (NCT03577028)	July 31, **2018**	Recruiting
	AMG160		PSMA	HLE-BiTE	Amgen	Prostate cancer	**phase I** (NCT03792841)	February 5, **2019**	Recruiting
	MOR209		PSMA	ADAPTIR	Aptevo Therapeutics	Prostate cancer	**phase I** (NCT02262910)	January **2015**	Completed
	BAY2010112		PSMA	BiTE	Bayer	Prostate cancer	**phase I** (NCT01723475)	November 2, **2012**	Completed
	Ertumaxomab		HER2	Triomab	Trion	Breast cancer	**phase II** (NCT00351858)	July **2006**	Terminated
	GBR1302		HER2	BEAT	Glenmark Pharmaceuticals	Solid tumor	**phase I** (NCT02829372)	May **2016**	Terminated
	M802		HER2	YBODY	YZYBio	Solid tumor	**phase I** (NCT04501770)	September 17, **2018**	Recruiting
	RG6194		HER2	NA	Genentech	Solid tumor	**phase I** (NCT03448042)	June 6, **2018**	Recruiting
	PF06671008		P-cadherin	DART/KIH	MacroGenics	Solid tumor	**phase I** (NCT02659631)	April 28, **2016**	Terminated
	MGD007		gpA33	DART	MacroGenics	Colorectal cancer	**phase I/II** (NCT03531632)	June 4, **2018**	Active, not recruiting
	MGD009		B7H3	DART/KIH	MacroGenics	Solid tumor	**phase I** (NCT02628535)	September **2015**	Terminated
	AMG757		DLL3	HLE-BiTE	Amgen	SCLC	**phase I** (NCT03319940)	December 26, **2017**	Recruiting
	REGN4018		MUC16	NA	Regeneron	Solid tumor	**phase I** (NCT03564340)	May 21, **2018**	Recruiting
	AMG596		EGFRvIII	BiTE	Amgen	Glioblastoma	**phase I** (NCT03296696)	April 18, **2018**	Active, not recruiting
	ERY974		GPC3	ART-Ig	Chugai	Gastric cancer and esophageal squamous cell carcinoma	**phase I** (NCT02748837)	August **2016**	Completed
	Tidutamab		SSTR2	Xmab	Xencor	Solid tumor	**phase I** (NCT03411915)	January 22, **2018**	Recruiting
	huGD2-BsAb		GD2	NA	Y-mAbs	Neuroblastoma	**phase I/II** (NCT03860207)	February 22, **2019**	Recruiting
	MGD014		HIV-1 Env	DART/KIH	Macrogenics	HIV-1 infection	**phase I** (NCT03570918)	September 25, **2018**	Recruiting
	AFM13	**CD16**	CD30	TandAbs	Affimed	Hodgkin’s Lymphoma	**phase II** (NCT04101331)	November 13, **2019**	Recruiting
	GTB-3550		CD33	TriKE	GT Biopharma	Hematological malignancies	**phase I/II** (NCT03214666)	January 1, **2020**	Recruiting
	TG-1801	**CD47**	CD19	κλ body	TG Therapeutics	B cell lymphoma	**phase I** (NCT03804996)	March 5, **2019**	Recruiting
**Blocking of immune checkpoints**	XmAb23104	**PD1**	ICOS	Xmab	Xencor	Solid tumor	**phase I** (NCT03752398)	May 1, **2019**	Recruiting
	AK104		CTLA4	ITab	Akesobio AU	Adenocarcinoma of the stomach or gastroesophageal junction	**phase II** (NCT04380805)	July 15, **2020**	Recruiting
	MGD019		CTLA4	DART	Macrogenics	Solid tumor	**phase I** (NCT03761017)	December 12, **2018**	Recruiting
	XmAb20717		CTLA4	Xmab	Xencor	Solid tumor	**phase I** (NCT03517488)	July 10, **2018**	Recruiting
	MEDI5752		CTLA4	DuetMab/KIH	AstraZeneca	Solid tumor	**phase I** (NCT04522323)	August 5, **2020**	Recruiting
	MGD013		LAG3	DART	Macrogenics	Solid tumor	**phase II/III** (NCT04082364)	September 30, **2019**	Recruiting
	RG7769		TIM3	CrossMab/KIH	Roche	Solid tumor	**phase I** (NCT03708328)	October 15, **2018**	Recruiting
	LY3434172		PDL1	KIH	Eli Lilly	Solid tumor	**phase I** (NCT03936959)	May 24, **2019**	Active, not recruiting
	FS118	**PDL1**	LAG3	NA	F-Star	Solid tumor	**phase I** (NCT03440437)	April 16, **2018**	Active, not recruiting
	KN046		CTLA4	CRIB	Alphamab	Solid tumor	**phase II** (NCT04469725)	August 31, **2020**	Not yet recruiting
	LY3415244		TIM3	NA	Eli Lilly	Solid tumor	**phase I** (NCT03752177)	November 22, **2018**	Terminated
**Blocking of inflammatory factors**	ATN103	**HSA**	TNF	Nanobody	Ablynx	RA	**phase II** (NCT01063803)	February **2010**	Completed
	ALX0061		IL6R	Nanobody	Ablynx	RA	**phase II** (NCT02518620)	July **2015**	Completed
	ALX0761		IL17A/F	Nanobody	Ablynx	Psoriasis	NA	NA	NA
	ALX0141		RANKL	Nanobody	Ablynx	Osteoporosis	NA	NA	NA
	SAR156597	**IL-13**	IL4	DVD-Ig	Sanofi	Idiopathic pulmonary fibrosis	**phase II** (NCT02921971)	November 23, **2016**	Completed
	GSK2434735		IL4	DVD-Ig	GlaxoSmithKline	Asthma	**phase II** (NCT01563042)	February 13, **2012**	Completed
	RG7990		IL17	NA	Genentech	Asthma	**phase I** (NCT02748642)	April 7, **2016**	Completed
	AMG570	**BAFF**	ICOSL	NA	Amgen	RA	**phase II** (NCT04058028)	February 19, **2020**	Active, not recruiting
	LY3090106		IL-17A	IgG4-(scFv)2	Eli Lilly	Sjogren syndrome	**phase I** (NCT03736772)	November 19, **2018**	Completed
	MEDI7352	**NGF**	TNF	scFv-Fc-Fab	Medimmune	Osteoarthritis	**phase II** (NCT03755934)	November 19, **2018**	Active, not recruiting
**Blocking of dual signal pathways**	ABT165	**EGFR**	DLL4	DVD-Ig	AbbVie	Solid tumor	**phase II** (NCT03368859)	February 19, **2018**	Completed
	ABL-001		DLL4	IgG-scFv	ABL Bio	Solid tumor	**phase I** (NCT03292783)	September 18, **2017**	Recruiting
	Navicixizumab		DLL4	NA	Celgene/Oncomed	Solid tumor	**phase I** (NCT03030287)	December **2016**	Completed
	RG7221		Ang-2	CrossMAb	Roche	Colorectal cancer	**phase I** (NCT01688206)	October 31, **2012**	Completed
	BI 836880		ANG2	nanobody	Ablynx	NSCLC	**phase I** (NCT02689505)	April 4, **2016**	Completed
	RO5520985		ANG2	CrossMab/KIH	Roche	Solid tumor	**phase I** (NCT02715531)	April 6, **2016**	Active, not recruiting
	JNJ-61186372		c-MET	DuoBody	Janssen R&D	NSCLC	**phase I** (NCT04077463)	September 4, **2019**	Recruiting
	EMB01		c-MET	FIT-Ig	Epimab Biotherapeutics	Solid tumor	**phase I** (NCT03797391)	December 13, **2018**	Recruiting
	MCLA-158		LGR5	Biclonics	Merus	Colorectal cancer	**phase I** (NCT03526835)	May 2, **2018**	Recruiting
	MCLA-128	**HER2**	HER3	NA	Merus	Breast cancer	**phase II** (NCT03321981)	January 15, **2018**	Active, not recruiting
	KN026		HER2	CRIB	Alphamab	Solid tumor	**phase II** (NCT04521179)	October 1, **2020**	Not yet recruiting
	MBS301		HER2	KIH	Beijing Mabworks Biotech	Solid tumor	**phase I** (NCT03842085)	April 11, **2019**	Recruiting
	ZW25		HER2	Azymetric	Zymeworks	Solid tumor	**phase II** (NCT04513665)	August 12, **2020**	Recruiting
	MP0274		HER2	NA	Molecular Partners AG	Solid tumor	**phase I** (NCT03084926)	August 8, **2017**	Active, not recruiting
	RG7386	**FAP**	DR5	CrossMAb	Roche	Solid tumor	**phase I** (NCT02558140)	October 11, **2015**	Completed
	MGD010	**CD32B**	CD79B	DART	MacroGenics	Autoimmune disease	**phase I** (NCT02376036)	February **2015**	Completed
	RG7992	**FGFR1**	KLB	KIH	Genentech	II diabetes	**phase II** (NCT04171765)	September 30, **2020**	Recruiting
	MEDI3902	**PsI**	Pcrv	scFv-Fc-Fab	Medimmune	Pneumonia	**phase II** (NCT02696902)	March 25, **2016**	Completed
	BI1034020	**Aβ40**	Aβ42	IgG assembled from half-Abs	Boehringer ingelheim	Alzheimer disease	**phase I** (NCT01958060)	October **2013**	Terminated
	Emicizumab	**FIXa**	FX	ART-Ig	Roche	Hemophilia A	Approved	NA	Approved

Data come from clinical trials, IMGT and referenced 128 and 140.

## Applications of BsAbs

BsAbs are widely used in both diagnosis and therapy. In terms of diagnosis, BsAbs can be combined with HRPO; be used in pre-targeting strategies to assist in clinical diagnosis; and provide better imaging for the early detection, diagnosis, and treatment of cancer. In terms of therapy, the main strategy is to precisely target and reactivate immune cells, help regulate the activation of immune cells, fine-tune the fate and function of immune cells, improve the tolerance of immune cells, and promote a return to immune homeostasis. In addition to tumors, BsAbs can treat other diseases, such as hemophilia A, diabetes, Alzheimer’s disease, and ophthalmological diseases.

### Applications in Diagnostics

#### Clinical Diagnosis

Because BsAbs can bind to specific antigens and specific detection sites simultaneously, their design is flexible, sensitive, and convenient. They can reduce the harmful effects of chemical modification of enzymes or antibodies, simplify detection, and help detect infectious bacterial and viral diseases and diagnose cancer (125). Lipoarabinomannan (LAM), an important non-protein antigen of the cell wall of tuberculosis bacteria, is found in various bodily fluids of infected patients. BsAbs that target LAM and HRPO can be used for the rapid detection of tuberculosis bacteria. An evaluation of 21 stored clinical serum samples showed that the BsAb assay has 100% specificity and 64% sensitivity, furthermore, results can be obtained in 2 h, in comparison traditional laboratory culture requires 2 to 6 weeks (126). Another BsAb targeting human red blood cells (RBCs) and hepatitis B virus surface antigen (HBsAg) is used to detect HBsAg in blood samples. In an enzyme-linked immunosorbent assay, this BsAb showed specific binding to RBCs and HBsAg. Compared with conventional immunoassays, this method has a specificity of 100% and sensitivity of 97.7% and is simple and quick; no special equipment or training is needed (127, 128). *Escherichia coli* O157:H7 is a serious human pathogen that causes hemorrhagic colitis and hemolytic uremic syndrome. Quadroma P126 targets O157 and HRPO. *E. coli* O157:H7 can be quickly detected by ELISA with Quadroma P126. As above, the principal action of this BsAb is to target *Bordetella pertussis* LPS and HRPO with a detection limit of 5 CFU (129). *Staphylococcus aureus* causes wound infection. BsAbs target HRPO and *S. aureus* thermal nuclease (TNase) to enable detection of the presence of *S. aureus (* 130). Finally, BsAb targeting of the nuclear plasmid protein of the SARS coronavirus immunoassay antigen is superior to that by MoAb in terms of specificity, sensitivity, and signal strength while maintaining a minimal signal background (131).

#### Medical Imaging

TF2 is constructed *via* the dock-and-lock platform, which can simultaneously recognize the histamine succinate glycine (HSG) motif and CEA (132). IMP288 is a divalent HSG hapten that can be labeled with a variety of radionuclides (^90^Y, ^177^Lu, ^111^In, ^124^I, ^18^F, ^68^Ga) (133). In clinical diagnosis, ^18^F fluorodeoxyglucose positron emission tomography (FDG-PET) can identify cancer and new potential lesions. However, FDG-PET has limited sensitivity to tumors smaller than 1 cm and lacks specificity for inflammatory or infectious lesions (134). Therefore, pre-targeted detection based on BsAbs is used in clinic. First, a BsAb that specifically recognizes both tumor antigens and small molecule polypeptides is injected into patient. Then, a small radiolabeled molecule is injected into the tumor location and combined with the pre-targeted BsAb when the concentration of BsAb in the blood increased to achieve blood clearance. The tumor is assessed based on radioactive radiation, and the excess radiolabeled small molecule is excreted. This method has high binding specificity, sensitivity, and safety. Pre-targeting often uses the positron nuclide ^68^Ga, which has a short half-life (135). In one study, the sensitivity of pre-targeting treatment with TF2 for tumor detection was 67% in a pre-targeting group but only 31% in a control group. For tumors smaller than 200 mg, sensitivity was 44% in the pre-targeting group and 0% in the control group (136). A TF2 immuno-PET and ^68^Ga phase II trial (NCT02587247) showed that pre-target immunization with anti-TF2 and ^68^Ga-labeled IMP288 was safe and feasible; 11 patients had no adverse reactions. Based on lesion analysis, the sensitivity, specificity, positive predictive value, and negative predictive value were 76%, 67%, 87%, and 33%, respectively, for the control group (FDG-PET) and 88%, 100%, 100%, and 67% for the pre-target immune group, which shows that pre-targeting with BsAbs has good diagnostic value (137). Early detection and diagnosis of pancreatic cancer remains a major clinical challenge. The anti-MUC1 MoAb PAM4 is highly specific for pancreatic adenocarcinoma. Comparison of TF10 (PAM4×HRPO) pre-targeting with that of directly radiolabeled PAM4, *via* imaging examination of the TF10 pre-targeting group 3 h after the radiolabeled peptide was injected showed a large amount of uptake in the tumor; however, none was detected in normal tissue. Therefore, TF10 pre-targeting can provide better imaging for early detection, diagnosis, and treatment of pancreatic cancer (138, 139).

### Applications in Therapy

#### Tumors

Over 86% of the BsAbs developed to date are used in cancer treatment (140). During targeted cancer therapy, the half-life of BsAbs changes with their molecular size. For some BsAbs of small molecular size, such as those on the BiTE platform, it is necessary to increase their half-life, for example by adding a section of Fc to avoid multiple infusions. Some nanobodies extend the half-life by conjugation. For example, adding a human serum albumin binding domain can enlarge the molecular size and promote the localization of BsAbs (141).

**Figure 2:**
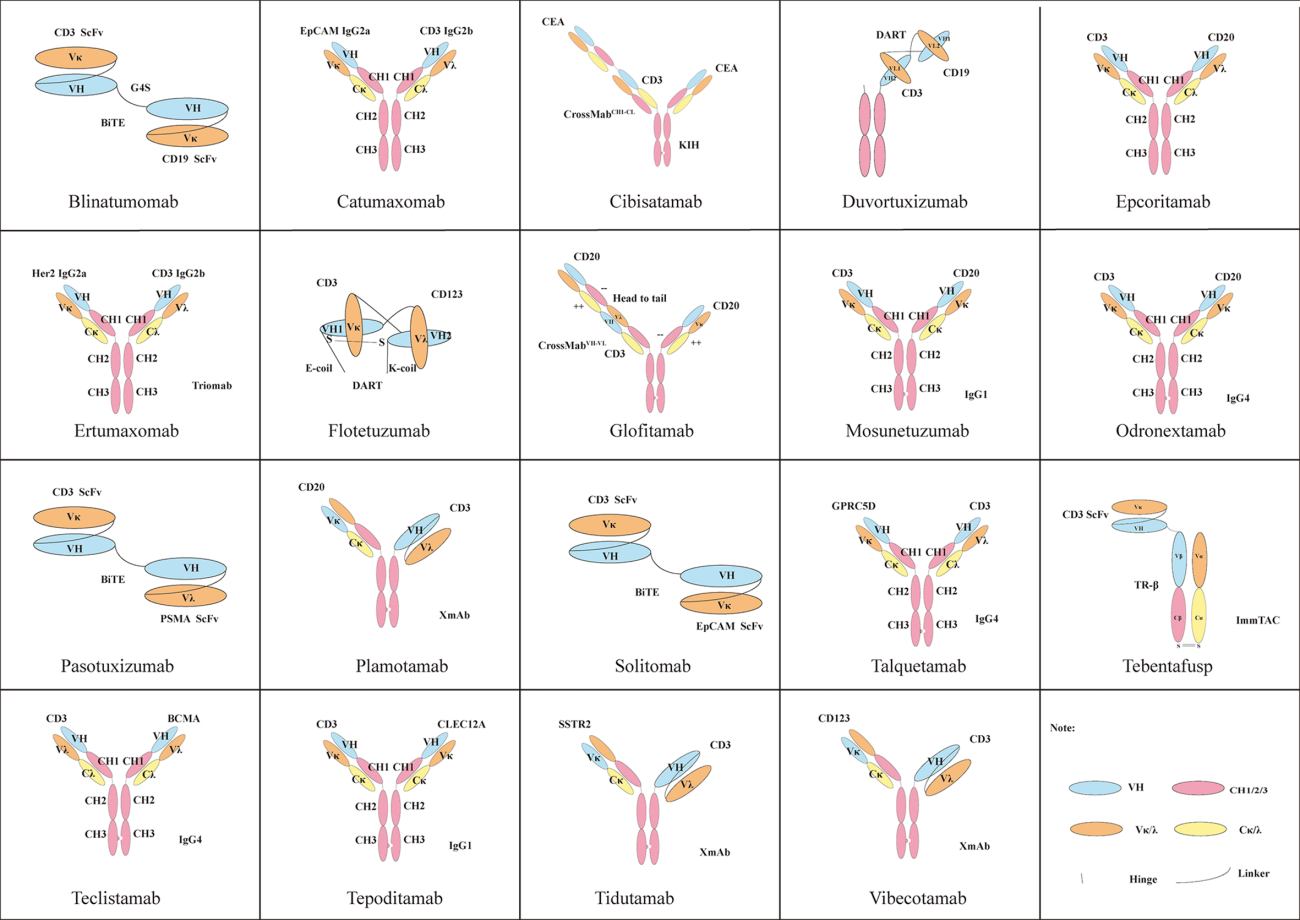
Some classic structures of BsAbs with CD3 as one of their targets.

BsAbs that use multivalent states targeting CD3 demonstrate a better tumor killing effect than 1 + 1, such as the TCB platform. Targeting different positions of antigens and different affinities can also cause huge differences in effect (142). The high CD3 affinity of BsAb is not necessary, because the low-affinity BsAb of CD3 shows the same killing effect *in vivo* and has better safety and good tolerance than the high-affinity BsAb (CD3×CLL1) of CD3 (143). Therefore, BsAbs often use a low-affinity antibody for CD3 and a high-affinity antibody for TAA (144, 145). TAAs with different levels of expression required the selection of antibodies with different affinities. Different platforms have different effects: for example, compared with scFv-based BsAbs, Fab-based BsAbs have better biophysical properties. In addition, Fab-based BsAbs can not only duplicate the activity of their scFv counterparts but also have unique biological activity (146). **Figure 2** shows the structures of BsAbs with CD3 as one of their targets

#### Diabetes

RG7992 (BFKB8488A) targets fibroblast growth factor receptor 1 (FGFR1) and coreceptor β klotho (KLB) (147, 148). FGFR1 improves insulin sensitivity, ameliorates liver steatosis, and accelerates weight loss (149, 150). Although FGFR1 is widely expressed in many cells, β-klotho is restricted to the liver and adipose tissues (151, 152). Therefore, RG7992 can avoid extensive FGFR activation and provide clinical benefits for patients with obesity and diabetes. A clinical trial (phase I: NCT02593331) evaluated the safety, tolerability, and pharmacokinetics of BFKB8488A in overweight and obese participants who may have had insulin resistance.

#### Alzheimer’s Disease

BI 1034020 targets two different epitopes of Aβ (Aβ40 and Aβ42) with high affinity (153). Its purpose is to reduce the level of free Aβ peptide in plasma and thus prevent the formation of new Aβ plaques and clear existing plaques (153). Furthermore, because the antibody targeting the transferrin receptor (TfR) can cross the blood-brain barrier(BBB) of mice through TfR receptor-mediated cell transduction, BsAbs that target TfR and β-secretase 1(BACE1) can cross the BBB and reduce brain Aβ in a TfR affinity-dependent manner (154, 155). Injecting anti-TfR/BACE1 BsAb into monkeys has been shown to reduce Aβ levels in cerebrospinal fluid and brain tissue in a concentration-dependent manner (156).

In terms of crossing the BBB, ANG4043 provides a new strategy to target Angiopep-2 (An2) and HER2. An2 can bind to low-density lipoprotein-like receptor 1 (LRP1), which effectively crosses the BBB ​​through receptor-mediated endocytosis and is taken up by cells expressing LRP1 (157). Preclinical evidence on ANG4043 shows that it not only treats HER2-positive brain metastasis but is also helpful in treating various brain tumors and other diseases of the central nervous system.

#### Pneumonia and Eye Diseases

The abuse of antibiotics, has led to an increase in bacterial resistance, and *Pseudomonas aeruginosa* has emerged as the main culprit behind clinical lung infections (158). In the rabbit lung infection model, MEDI3902 against the type 3 secreted protein (PcrV) and Psl exopolysaccharide has a protective effect in prevention and treatment. In animals treated with MEDI3902, *Pseudomonas aeruginosa* was found to be greatly reduced, and the organ burden and the expression of pro-inflammatory mediators in lung tissue were also significantly decrease (159, 160). A phase I (NCT02255760) dose escalation study of MEDI3902 showed that the severity of treatment-emergent adverse events (TEAE) was mild or moderate at all doses, and the serum anti-cytotoxicity and opsonizing phagocytic cell killing activity were found to be related to the serum concentration of MEDI3902. The results of the phase I study of MEDI3902 in healthy people proved its safety and effectiveness in *Pseudomonas aeruginosa* pneumonia (161).

Neovascular age-related macular degeneration (nAMD) is the main cause of vision loss in the elderly (162). The current standard of care for the treatment of nAMD is to inhibit VEGF (163). ANG-2 is a growth factor that plays a key role in vascular homeostasis, angiogenesis and vascular permeability. Faricimab (RG7716) targets at VEGF-A and ANG-2 shows greater efficacy than anti-VEGF-A alone in a laser-induced non-human primate model (164). The results of a phase I trial of RG7716 for nAMD showed no dose-limiting toxicity in single or multiple dose groups. In the combined single-dose groups and in the 6-mg multiple-dose group, best-corrected visual acuity (BCVA) increased from baseline to 28 days after the last dose administration by a median of 7 letters (range, 0–18 letters; n = 11) and 7.5 letters (range, 3–18 letters; n = 6). RG7716 is well tolerated and shows overall good safety (165).

### BsAbs ON THE MARKET

**Figure 3:**
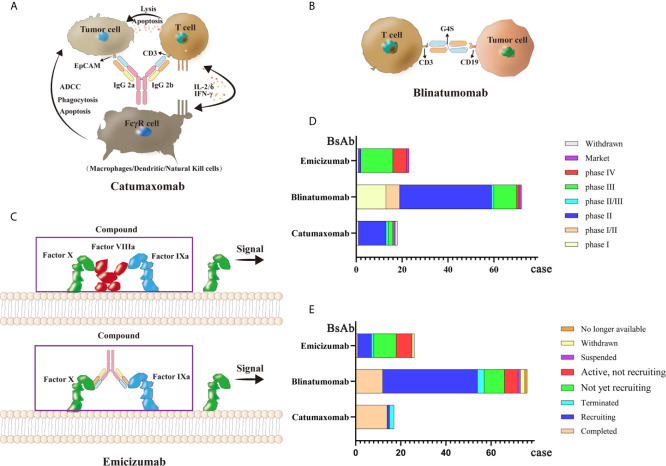
Mechanisms and clinical study of three market BsAbs: **(A)** Catumaxomab **(B)** Blinatumomab **(C)** Emicizumab **(D)** phase of three market BsAbs **(E)** status of three market BsAbs.

Currently, there are only three commercially available BsAbs. Catumaxomab, which was first approved in 2009, treats malignant ascites caused by malignant solid tumors by targeting both EpCAM and CD3. Then in 2014, Amgen’s blinatumomab was approved by the FDA to treat relapsed or refractory precursor B-cell acute lymphoblastic leukemia(ALL) with CD3/CD19 dual targets (166). Finally, Roche’s emicizumab, which was approved in 2017, acts on factor FIXa/FX through the formation of a protein complex to treat bleeding due to hemophilia A (167–169). Because of the company’s strategy, catumaxomab was withdrawn from the European Union market in 2017. Thus, only blinatumomab and emicizumab have been marketed globally. Clinical trials for these three antibodies are ongoing. A total of 77 clinical research projects focuses on blinatumomab; most clinical trials are currently in phase II, while most clinical trials of emicizumab are in phase III. Remarketing of catumaxomab has also begun. Structural diagrams of the three BsAbs and related clinical information are shown in **Figure 3**.

### Catumaxomab

Catumaxomab is a *Mus musculus* hybrid *Rattus norvegicus* Sprague Dawley trifunctional BsAb. The variable region of catumaxomab specifically targets the EpCAM antigen on tumor cells and the CD3 antigen on T cells. Targeting the CD3 antigen can stimulate T cells to secrete cytokines to eliminate tumor cells (170). The Fc segment can recruit Fc receptor-containing immune cells such as macrophages, dendritic cells (DCs), and natural killer cells (NKs) (171, 172). Catumaxomab shows good clinical efficacy with relatively safe and controllable side effects in patients with malignant ascites. A phase II (NCT00326885) clinical trial was conducted to assess the effectiveness and safety of catumaxomab in patients with chemotherapy-refractory ovarian cancer and recurrent malignant ascites. Among 40 screened patients, 32 treated patients (80%) (28 cases of ovarian cancer, and four cases of primary peritoneal cancer) received four 3-h intraperitoneal catumaxomab infusions (10, 20, 50, and 150 μg within 10 days). Seven patients (23%) achieved the primary end point of at least a four-fold increase in the puncture-free interval (PuFI) relative to the pretreatment interval. The median PuFI was prolonged two-fold while the median TTPu was prolonged four-fold. An effective reduction in the formation of ascites was also observed (173, 174). The most frequent adverse reactions during catumaxomab treatment are fever, nausea, fatigue, chills, abdominal pain, elevated liver enzymes and lymphopenia. However, these adverse events are predictable, relatively controllable, and mostly reversible (175–177).

### Blinatumomab

Blinatumomab acts on CD3/CD19 dual targets to treat Philadelphia chromosome-negative precursor B-cell ALL (178, 179). It is the first BsAbs drug approved by the FDA to treat leukemia cells through human T cells. Patients with ALL who are cured always have some measurable residual disease (MRD), which often causes a relapse (180, 181). After blinatumomab treatment, a certain degree of complete remission (CR) can be achieved (182–184). The main side effects of blinatumomab are fever, headache, edema, nausea, tremor, neutropenia, thrombocytopenia, and elevated transaminase (168, 185). A phase II (NCT01209286) trial was performed to determine clinical activity in patients with relapsed or refractory B precursor ALL. Thirty-six patients were treated with blinatumomab in cycles of 4-week continuous infusion; this single-arm exploratory study reported a CR or CR with partial hematological (CRh) recovery rate of 69%, with 88% of the responded patients achieving MRD response. Median overall survival (OS) was 9.8 months, and the median recurrence-free survival (RFS) was 7.6 months. The most frequent adverse reactions were fever (81%), fatigue (50%), headache (47%), tremor (36%), and leukopenia (19%), These medical events were resolved clinically (186). In another phase II clinical trial (NCT01466179), 63 out of 189 (33%) patients achieved CR and 18 (10%) patients achieved CRh. The most common adverse events were febrile neutropenia (48 cases, 25%) and anemia (27 cases, 14%) (187). Further, a phase I/II study of blinatumomab (NCT01471782) showed that of 70 patients, 27 (39%) achieved CR within the first two cycles and 14 (52%) achieved complete MRD response (188). In sum, blinatumomab has shown encouraging clinical effects.

### Emicizumab

Emicizumab is used to treat patients with congenital factor VIII deficiency (189). When healthy people bleed, factor FIXa and factor FX are gathered by factor VIII to form a coagulation complex. However, because they lack coagulation factor VIII, patients with hemophilia cannot form this coagulation protein (190, 191). Emicizumab acts as a bridge, connecting factor FIXa and factor FX together, and has a hemostatic effect (192). The original factor VIII drug has low subcutaneous bioavailability, a short half-life, and poor pharmacokinetics; therefore, frequent intravenous injection is required. Emicizumab has high subcutaneous bioavailability and a half-life of more than two weeks (167), hence its use as the main drug for treating hemophilia A. In a phase III clinical trial (NCT02622321), participants who had received episodic treatment with bypassing agents (BPA) before trial were divided into group A, receiving subcutaneous emicizumab prophylaxis (35 participants) and group B, without receiving emicizumab prophylaxis (18 participants). Patients in group A showed an annualized bleeding rate of 2.9 events, while those in group B reported 23.3 events (193). Another clinical trial (NCT02847637) showed that the bleeding rate of hemophilia A patients who received a subcutaneous injection of emicizumab per week (group A) or every 2 weeks (group B) decreased significantly compared with that of patients who received no preventive measures (group C). According to this study, emicizumab therapy led to a significantly lower bleeding rate than the previously used factor VIII prophylaxis (194).

## Conclusions

Progress in the field of antibody or protein engineering and recombinant DNA technology have led to the establishment of different platforms to generate BsAbs. These platforms have been applied to generate different structures of BsAbs that target different molecules or different epitopes of the same molecule. These different molecules could be either specifically from tumor cells, or from immunocytes, which enhance the anti-tumor efficacy of BsAbs. These different structures of BsAbs exert differing activity according to the status of different diseases. Therefore, the most optimal structures of BsAbs can be selected for the treatment of particular diseases. Cancers are the most common diseases for which BsAbs are developed. BsAbs have been also used in other kinds of diseases, as well as in molecular imaging for diagnosis. Three BsAbs have been approved for marketing in treatment of cancers and hemophilia A. Furthermore, 110 BsAbs have been tested in clinical trials at different stages. The future development of BsAbs will involve the development and use of new targets, new combinations, new platforms, and new geometric configurations as well as combined treatments with traditional biological drugs, other forms of immunotherapy, physical and chemical therapies. BsAbs may become a new focus of future medical and biological research and development.

## Author Contributions

JM, YM, YX, YQ, WZ, YH, and JS wrote the manuscript. MT, YX, and CQ reviewed and edited the manuscript before submission. JM prepared the figures and tables. All authors contributed to the article and approved the submitted version.

## Funding

This work was supported by National Key Research and Development Program (2016YFC1303405), (SQ2020YFF0401839), and National Natural Science Foundation of China (91959206).

## Conflict of Interest

Authors JS and YH were employed by the company Chongqing Precision Biotech Co., Ltd. Authors YQ, WZ, MT, and YX were employed by the company Chongqing Institute of Precision Medicine and Biotechnology Co., Ltd.

The remaining authors declare that the research was conducted in the absence of any commercial or financial relationships that could be construed as a potential conflict of interest.
